# Factors and Mechanisms Involved in Acquired Developmental Defects of Enamel: A Scoping Review

**DOI:** 10.3389/fped.2022.836708

**Published:** 2022-02-24

**Authors:** Anne-Margaux Collignon, Jean-Noël Vergnes, Alice Germa, Sylvie Azogui, Sophie Breinig, Clémence Hollande, Anne-Laure Bonnet, Cathy Nabet

**Affiliations:** ^1^URP 2496 Laboratory Orofacial Pathologies, Imaging, and Biotherapies and Life Imaging Platform (PIV), Montrouge, France; ^2^Department of Odontology, AP-HP, Paris, France; ^3^Dental School Faculty, Paris University, Paris, France; ^4^Department of Epidemiology, Public Health, Prevention and Legislation, Dental Faculty, Toulouse University Hospital, Paul Sabatier University, Toulouse, France; ^5^Inserm UMR 1295 CERPOP, Toulouse University, Toulouse, France; ^6^CRESS, EPOPE Team, INSERM, INRA, Paris, France; ^7^Education and Health Practices Laboratory (LEPS) (EA 3412), UFR SMBH, Paris 13 University, Sorbonne Paris Cité, Bobigny, France; ^8^Pediatric and Neonatal Intensive Care Unit, CHU Toulouse, Toulouse, France; ^9^Department of Hepatology, Université de Paris Centre, Hôpital Beaujon, AP-HP, Paris, France

**Keywords:** enamel, defect, scoping review, acquired—etiology, protective factor, risk factor

## Abstract

**Background:**

Developmental Defects of Enamel (DDE) is a pathology of the teeth that can greatly alter the quality of life of patients (hypersensitivity, esthetic issues, loss of function, etc.). The acquired DDE may occur as a result of a wide range of acquired etiological factors and his prevalence of this pathology may reach up to 89.9%. The main objective of this research was to identify and analyze, in current literature, the factors related to acquired DDE, in order to propose a general theory about the mechanisms involved.

**Methods:**

The search of the primary literature was conducted until [December 31, 2021]. Our search strategy uses the Pubmed/MEDLINE database and was structured around 3 terms [“Development,” “Defect,” and “Enamel”]. To be included, references had to be primary studies, written in English. Exclusion criteria were reviews, *in vitro*, animal, genetic or archeology studies, and studies focused on clinical management of DDE. One hundred and twenty three articles were included in this scoping review: 4 Randomized clinical trials, 1 letter, 5 cases reports, 2 fundamentals studies, and 111 observational studies (33 Cross-sectional studies, 68 Cohort study and 10 Case-control study). The quality of evidence was assessed using the PEDro scale for clinical trials, the Newcastle-Ottawa scale for observational studies, and a published tool to assess the quality of case reports and case series.

**Results:**

A scoping review of the literature identified 114 factors potentially involved in acquired DDE. The most frequently encountered pathologies are those causing a disorder of calcium homeostasis or a perturbation of the ARNT pathway in mother or child. The link between the ARNT pathway and metabolism deficiency in uncertain and needs to be defined. Also, the implication of this mechanism in tissue impairment is still unclear and needs to be explored.

**Conclusions:**

By identifying and grouping the risk factors cited in the literature, this taxonomy and the hypotheses related to the mechanism allow health practitioners to adopt behaviors that limit the risk of developing aDDE and to set up a prevention of dental pathology. In addition, by reviewing the current literature, this work provides guidance for basic research, clinical studies, and literature searches.

## Introduction

Developmental Defects of Enamel (DDE) are defined as disturbances in hard tissue matrices and their mineralization that arise during odontogenesis (1) (from 16 weeks of gestation to the age of 5). This set of non-carious lesions can affect both primary and permanent teeth and negatively impacts the health of children. Short to long-term potential adverse effects may occur, such as tooth sensitivity, carious lesions, low self-esteem or stigma experiences, and social costs including children's absence from school (2).

Prevalence of acquired DDE may reach up to 89.9% (3). A large body of literature exists about risk factors for DDE; however, DDE have been studied in silos rather than comprehensively, which has led to a rather poor understanding of pathogenic mechanisms involved in their occurrence.

Factors that may interfere with the metabolic process of enamel formation and lead to DDE are traditionally dichotomized as hereditary or acquired. A complete meta-analysis on the hereditary factors has been recently published (4). Acquired DDE (aDDE) occur when the disturbance is sufficient to alter the regular growth pattern of the enamel tissue. Currently, the etiology is still unclear, perhaps because the literature is extensive and sometimes inconsistent.

The main objective of this research was to identify and analyze the factors related to acquired DDE, in order to propose a general theory about the mechanisms involved. To achieve this objective, we designed a research framework based on the scoping review methodology, synthesizing current scientific knowledge on the subject. This work aims to help improve prevention, treatment, and multidisciplinary management strategies.

## Methods

To combine the exploratory and systematic nature of our research, we followed the Arksey and O'Malley methodological framework for scoping reviews (5), and completed the PRISMA for Scoping review guidelines (see Supplementary Material 1). The search of the primary literature was conducted until [December 31, 2021]. We focused our search strategy on the Pubmed/MEDLINE database, considering it would include all the major factors identified by biomedical research. We structured our search strategy around 3 terms [“Development,” “Defect,” and “Enamel”], allowing for explosion search, and using the Boolean operator AND (see details in Supplementary Material 2). We did not use terms involving any risk factor for DDE, so as to scan the database broadly without preconceived notions about the factors we were looking for. The screening process was performed by one author (AMC).

To be included, references had to be primary studies, written in English. Exclusion criteria were determined iteratively by a team approach (AMC, JNV, CN), i.e., as the data were filtered, according to the nature of the studies discovered during the initial screening, in accordance with the methodology of scoping reviews (5). Thus, excluded articles were reviews, *in vitro*, animal, genetic or archeology studies, and studies focused on clinical management of DDE. Two raters (AMC, CN) independently assessed the quality of evidence, using the PEDro Scale for clinical trials, the Newcastle-Ottawa scale for observational studies, and a published tool for evaluating the quality of case reports and case series. Agreement on quality ratings of full- text articles was assessed for each, including references, and disagreements were resolved by a consensus-based discussion with a third rater (JNV). Finally, one author (AMC) completed the search strategy using a snowball approach to scour the references sections of all included articles (see details in Supplementary Material 3).

### Subsections Relevant for the Subject

**Figure 1:**
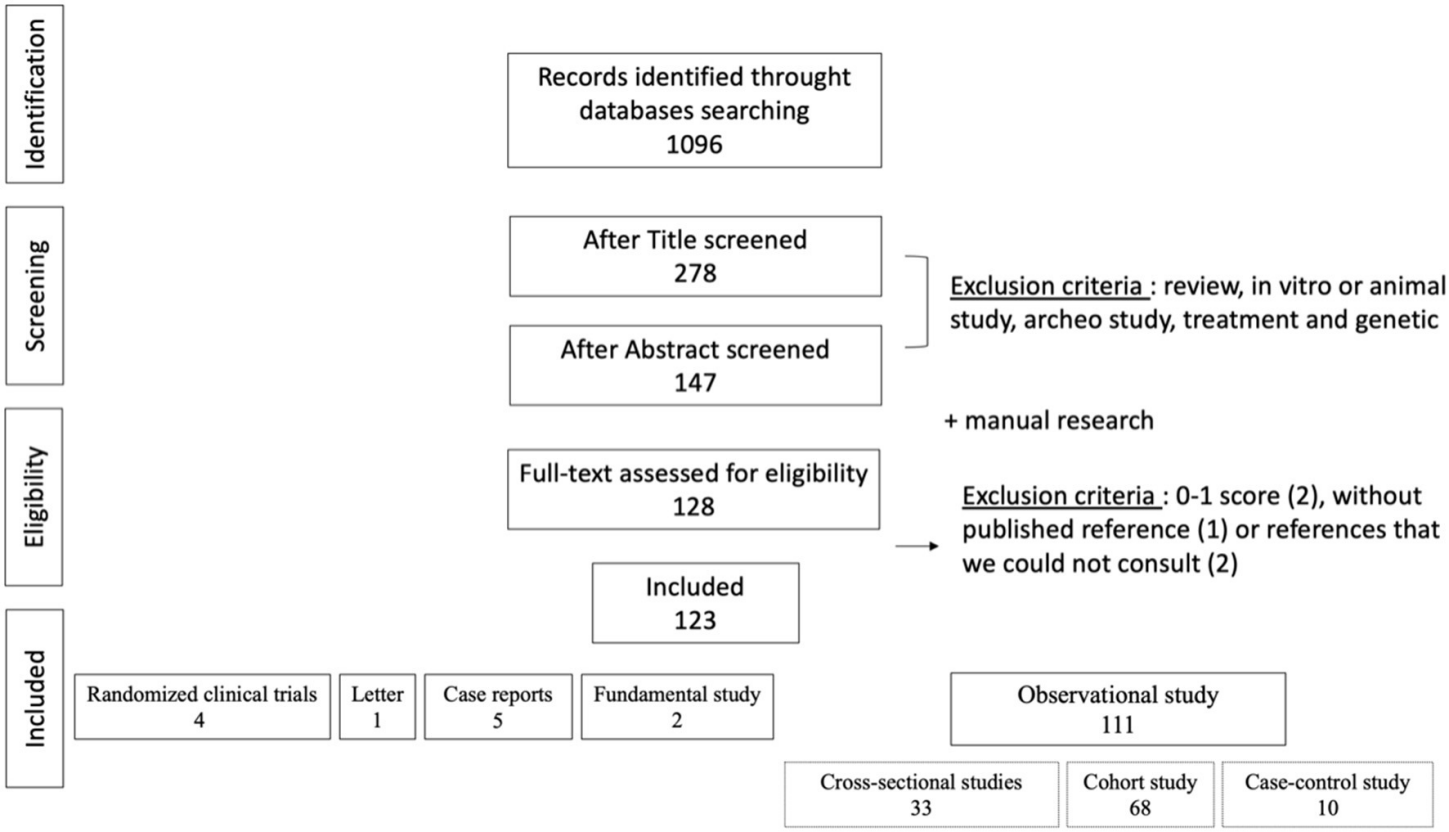
Flow chart of the research strategy.

The initial electronic database search represented a total of 1,096 articles. Considering exclusion criteria, 128 full-text articles were eligible for inclusion. We excluded 5 articles with a quality assessment score of 0 and without published references, or with references that could not be consulted. Finally, 123 articles were included in this scoping review (Supplementary Material 3). Figure 1 describes the flowchart for the entire process.

**Figure 2:**
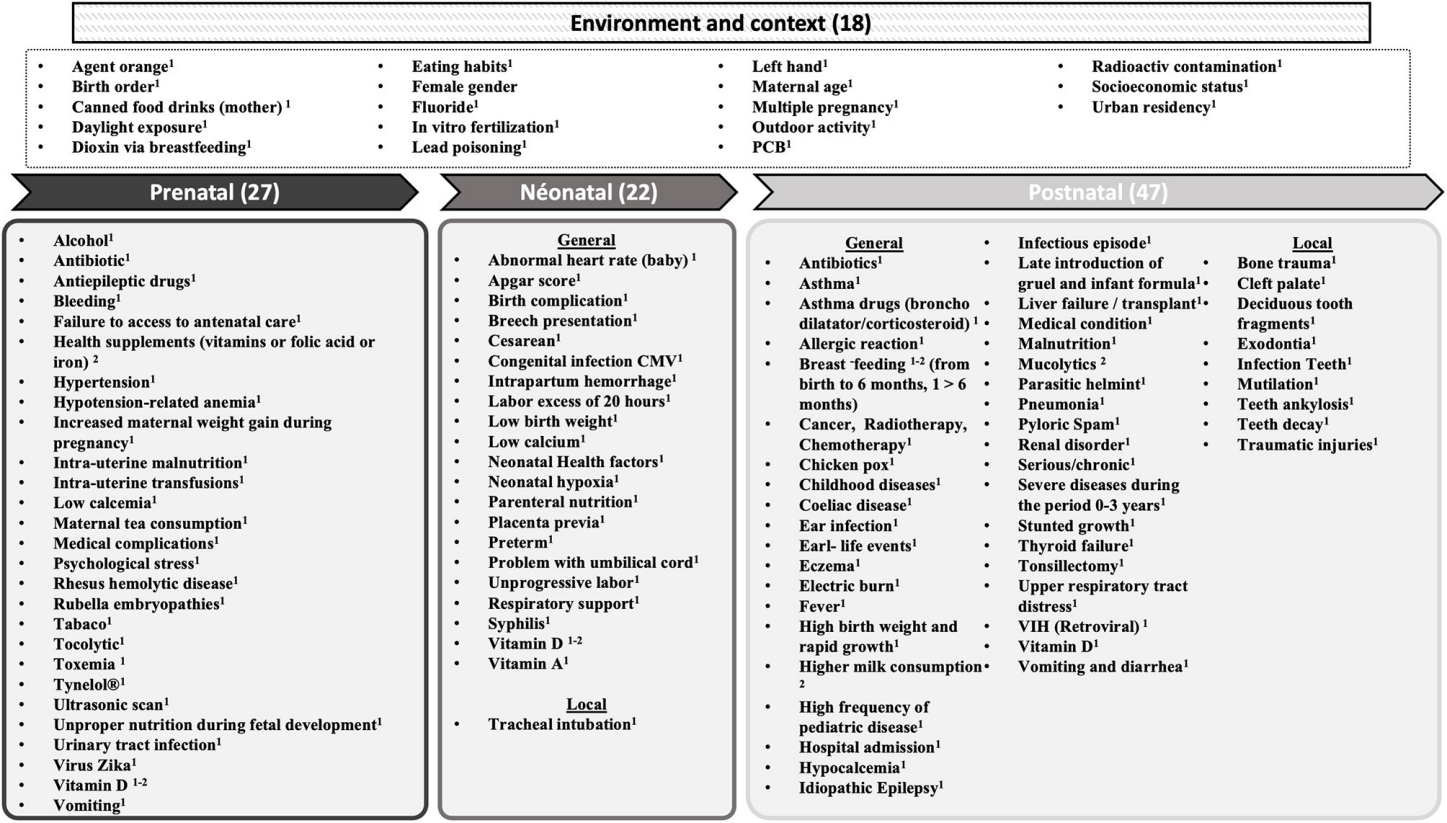
Factors cited in the literature related to aDDE, according to periods of damage. (–) number of factor, Risk factor^1^, Protective factor^2^.

**Figure 3:**
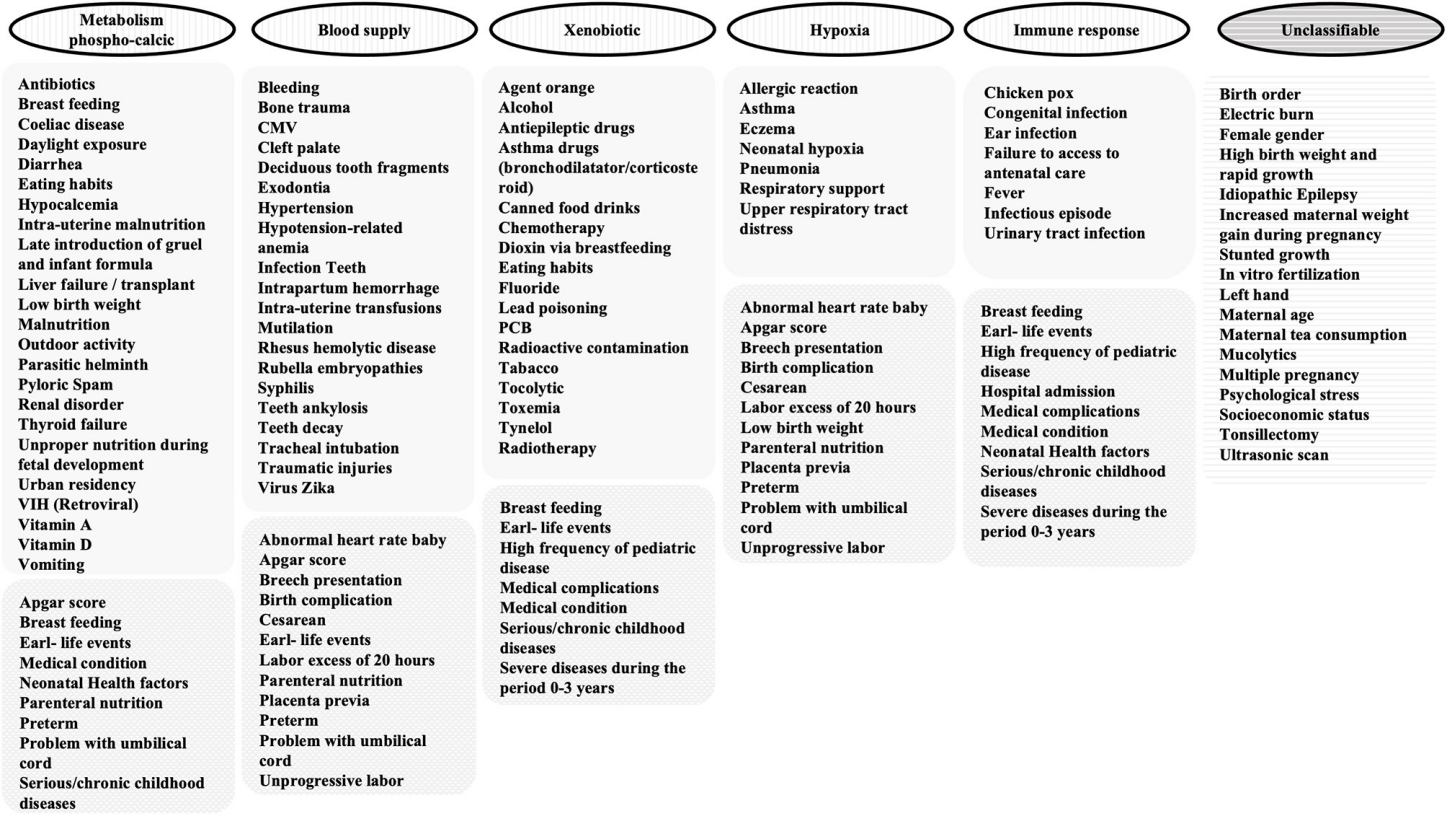
Clustering of aDDE risk and protector factors in 5 categories according/corresponding to the possible mechanisms.

Our explicative model about the mechanisms involved in acquired DDE (aDDE) is presented according to (1) a chronological timeframe of tooth development (Figure 2) and (2) putative biological mechanisms (Figure 3). All the factors are sorted alphabetically, without any prioritization.

**1) Chronological timeframe of tooth development**

As aDDE pathology occurs during a more or less extensive time-lapse of morphogenesis and biomineralization of the teeth and in agreement with Elzein et al. (6), we first classified the risk- or protective-related factors into three sections, corresponding to prenatal, neonatal, and postnatal periods. Some factors have been identified in each of these periods. Moreover, environmental (social context and geographic) factors were added (Figure 2).

#### Prenatal Risk and Protective Factors

Studies showed a higher frequency of aDDE among children: who suffered from intrauterine malnutrition (7, 8), improper nutrition during fetal development (9), and survivors after prenatal intra-uterine transfusions (for rhesus incompatibility for example) (10).

Medicals complications (11) or toxemia during pregnancy (12) are related to DDE. More specifically: ZIKV (13, 14), rubella embryopathy (15), deficiencies of vitamin D (16–18), low calcemia (16, 19), increased maternal weight gain and failure to access to antenatal care (20), urinary tract infection (21), gestational diabetes (16, 22, 23), bleeding (24), frequent vomiting (25), maternal psychological stress (26), hypotension-related anemia (26), hypertension (27), and frequent exposure to ultrasonic scans during the last gestational trimester (26). Maternal consumption of Tynelol during pregnancy (20), antibiotic (28) prenatal anti-epileptic drugs (29), tocolytic agent (30), alcohol (31, 32), tea (20), tobacco use (25, 33) were associated to DDE. A relationship between the number of cigarettes smoked per day and the prevalence of hypoplasia was described (27, 34). Conversely, several studies suggested that vitamin D supplementation (35–37) or health supplements (vitamins or folic acid or iron) (25) during pregnancy would be beneficial to prevent aDDE.

#### Neonatal Risk Factors

Neonatal health factors appeared to be associated with enamel defects (38). Delivery complications (39), non-progressive labor, umbilical cord issues and/or an abnormal heart rate of the baby (6), labor more than 20 hours, breech presentation, intrapartum hemorrhage, and placenta previa (12), cesarean delivery (12, 34) were associated with DDE.

Preterm birth could be considered as risk factor for aDDE (8, 12, 40–43) and low birth weight has been associated with DDE (7, 8, 34, 44–47) or the association preterm low birth weight children (48). A lower Apgar score was associated with a higher prevalence of enamel defects (34, 49–51).

Associations between vitamin D deficiencies (33), low calcemia (19, 52), low vitamin A levels (53), infections [syphilis (54) or CMV (55)] and DDE were also described.

Tracheal intubation (34, 43, 56) may cause local trauma and dental lesions associated with endotracheal intubation are asymmetrical. And even that the longer the duration of mechanical ventilation, the greater the chance of developing dental enamel defect (57). aDDE were more common in children who received parenteral nutrition during the neonatal period (34).

#### Postnatal Risk and Protective Factors

Early-life events (49), medical condition (26), severe diseases during the period 0–3 years (58), and high frequency of pediatric disease (21) appear to be associated with aDDE. This may involve serious/chronic childhood diseases (11, 14, 59), such as the following factors cited in the literature: vitamin D deficiencies (17), hypocalcemia (60), chicken pox (61), infectious episodes (38, 53), high fever (6, 62), hospital admission (40), ear infection (27, 63), renal disorders (64), liver failure/ liver transplant (65), thyroid dysfunction (both hypothyroidism and hyperthyroidism) (66), infantile eczema (33), tonsillectomy (67), intestinal disturbance [parasitic helminth infection (68) and pyloric spasm (63), celiac disease (69, 70), vomiting and diarrhea (61)], electric burns (71), idiopathic epilepsy (72), and the use of some drugs: antiretroviral therapy (24), antibiotic use (6, 14, 33, 73), specifically penicillins (74, 75) and cephalosporins (76).

Age of children (when perturbation happens) matters: first month (50), first year of life (40, 62), the first 2 years of the child's life (77) or the 3-year barrier are cited (39, 73).

aDDE are described in children surviving cancer (78–82) because of the disease itself, or anticancer therapy (83): antineoplastic agents (84–86), and/or radiotherapy (87). Dental abnormalities occur more frequently in patients who have undergone treatment during odontogenesis [different studies have proposed some ages: children younger than 1 (78), 3 (88), 5 (84, 89), or 6 (87) years].

The association of severe respiratory distress syndromes leading to oxygen deprivation (asthma, upper respiratory tract distress, pneumonia) and aDDE is reported by several studies (38, 39, 61, 77, 90, 91). More specifically, the severity (61) of asthma, asthma drugs (92) (bronchodilator/corticosteroid) or severe allergic reactions (91) are linked to a higher prevalence of aDDE.

Malnutrition is related to the prevalence of aDDE (28, 59, 68). Stunted growth (68, 93) seems to lead to enamel defects. Children with a high birth weight and rapid growth during their first year of life were more vulnerable to the occurrence of aDDE in their permanent dentition (3).

Higher milk consumption postnatally (94) and treatment with mucolytics (21) seemed to exert a protective effect against aDDE.

Breastfeeding was described as a protective factor for enamel defect development (95, 96) and children who were not breast-fed could be considered at risk for developing enamel defects (41, 94, 97). On the contrary, an association between aDDE and breastfeeding for more than 6 months, with late introduction of gruel and infant formula was found (98). The conclusion was that nutritional conditions during the first 6 months of life may influence the risk of developing severe demarcated opacities in the first permanent molars.

Cleft palate (CLP) is associated with aDDE (99, 100). The prevalence of dental abnormalities in CLP patients will depend on treatment protocol (101, 102) and that surgical repair will contribute to this defect (103).

Also, a correlation between aDDE on permanent teeth and pathology of the predecessors is suggested: retained deciduous tooth fragments (104), ankylosis (105), exodontia (106), traumatic injuries (107–111), periapical infection (16, 112), decays (113, 114), mutilation (115, 116), self-inflicted minor oral trauma among infants learning to handle and mouth objects (94).

#### Environment (Social Context and Geographic) Risk Factors

The prevalence of aDDE seems to be higher with younger maternal age (34, 97), in case of multiple pregnancy (12, 34), *in vitro* fertilization (14) and with Sibling birth order (26, 50).

Higher socioeconomic status was found to be a risk factor for aDDE (33). The rate rose when moving from the most deprived categories to the least deprived categories (117). Female gender (21) and Left -handers may be associated with hypoplastic defect (20).

Reduced exposure to daylight because of winter months (17, 96, 118), little outdoor activity (25, 118), Urban residency during a child's first 2 years (119) and eating habits (118) seem associated with enamel disturbances, possibly reflecting vitamin D status.

Chemical elements may cause an adverse effect on dental formation. An association appears to exist between aDDE and some toxins: radioactive contamination (120), dioxin exposure (121) (“Agent Orange”), chronic lead poisoning (122), and long- term exposure to PCBs (123) and excess fluoride (124).

Prolonged breast feeding (more than 8 months) may increase the risk of mineralization defects in healthy children (125–127), possibly because of environmental contaminants that interfere with tooth development (128). Maternal consumption of canned food and drinks during breastfeeding was associated with aDDE (6).

**2) Putative biological mechanisms**

The mechanisms involved in aDDE identified in the scoping review were regrouped into 5 categories according/corresponding to the possible mechanisms (Figure 3).

#### Phosphocalcic Metabolism

Metabolic stresses/disorder (18, 129) or hypocalcemia during enamel formation (130) may be associated with aDDE. More specifically, some pathologies identified in the scoping review may cause calcemia disorders and have a link with aDDE: chronic illness or malnutrition problems (131), vitamin D deficiency (16, 132), phosphate deficiency (130, 131, 133), thyroid dysfunction (130, 131), celiac disease (issue in calcium absorption) (134), diabetic disease (23), gestational diabetes (135), cancer (136) calcium metabolism (16, 130, 133), pediatric conditions (18), and antiretroviral therapy (HIV treatment) for children and adolescents because it leads to a reduced level of calcium (137).

#### Blood Supply

Local trauma due to CLP surgery (102), decay (because there is spread of inflammation to the underlying permanent teeth germs) (113) and virus (due to vascular changes leading to the lack of cellular nutrition) (138) perturbs blood supplies. Even if a possible direct cause due to the virus is not ruled out (139).

#### Xenobiotics

Teeth are most vulnerable and sensitive to the toxic effects of environmental chemicals and drugs during their development and before eruption into the mouth (140).

Exposure to dioxins is therefore not without danger for the dental organ and its consequences are closely linked to the stage of development of the tooth and the toxic dose received (141).

#### Hypoxia

Conditions such as neonatal hypoxia (16), severe allergies (91), asthma, and other respiratory diseases (129) can cause respiratory acidosis and abnormal oxygen levels (142) and may be associated with aDDE. Moreover, because ameloblasts (cells which secrete the enamel proteins) are highly sensitive to oxygen supply (77), oxygen deficiency was proposed as perturbing the mineralization of the enamel matrix (143).

#### Immune Response

Infections affect the immune system and cause systemic repercussions that can affect dental development (129, 144), especially by altering normal ameloblast function (30).

## Discussion

This scoping review which meets the objectives described in the Munn et al. (145) report made it possible to:

### Highlight the Current Literature and Better Understand the Needs

The research on factors related to aDDE is mainly supported by observational studies (Figure 1). Quality scores of the included studies were heterogeneous, ranging from 1/8 to 8/9, with a mean score of 4.8/8 and a median at 5/8 (Supplementary Material 3). One hundred and fourteen factors were cited in the literature. This allows a mapping chronological timeframe (Figure 2) and a classification by putative biological mechanisms (Figure 3).

Some factors are well-reported and have been the subject of review papers (not analyzed in this SC): severe respiratory distress syndromes (146), preterm birth (147), vitamin D deficiencies during childhood (148), cleft palate (149), syphilis (150), excess fluoride (151), renal disorders (133), diabetes type 1 (152), cancer therapy (153), organic pollutants (154), dioxin exposure (155), dietary components (156), drugs during pregnancy and the first year of life (157), and drugs during infancy (158); these do not always relay clear evidence between DDE and risk factors supporting our hypothesis of accumulation and synergy effects. Generalist reviews were found, but either did not identify as many factors in a systematic way (159), or focused on one type of DDE (160), dentition (161) or treatment (162). Even if our results are close/comparable to the recent published study (163) on a particular form of DDE affecting the 1st molar and permanent incisors (MIH), this taxonomy raise some lacks in the current literature. This SC opens research perspectives by pointing out that some factors could be the object of review and that conduct large-scale epidemiological studies could be beneficial to study potential associations of protective or risk factors.

### Work Around Available Evidence and Key Concepts in the Literature to Advance Hypotheses About Common Pathophysiological Mechanisms

**Figure 4:**
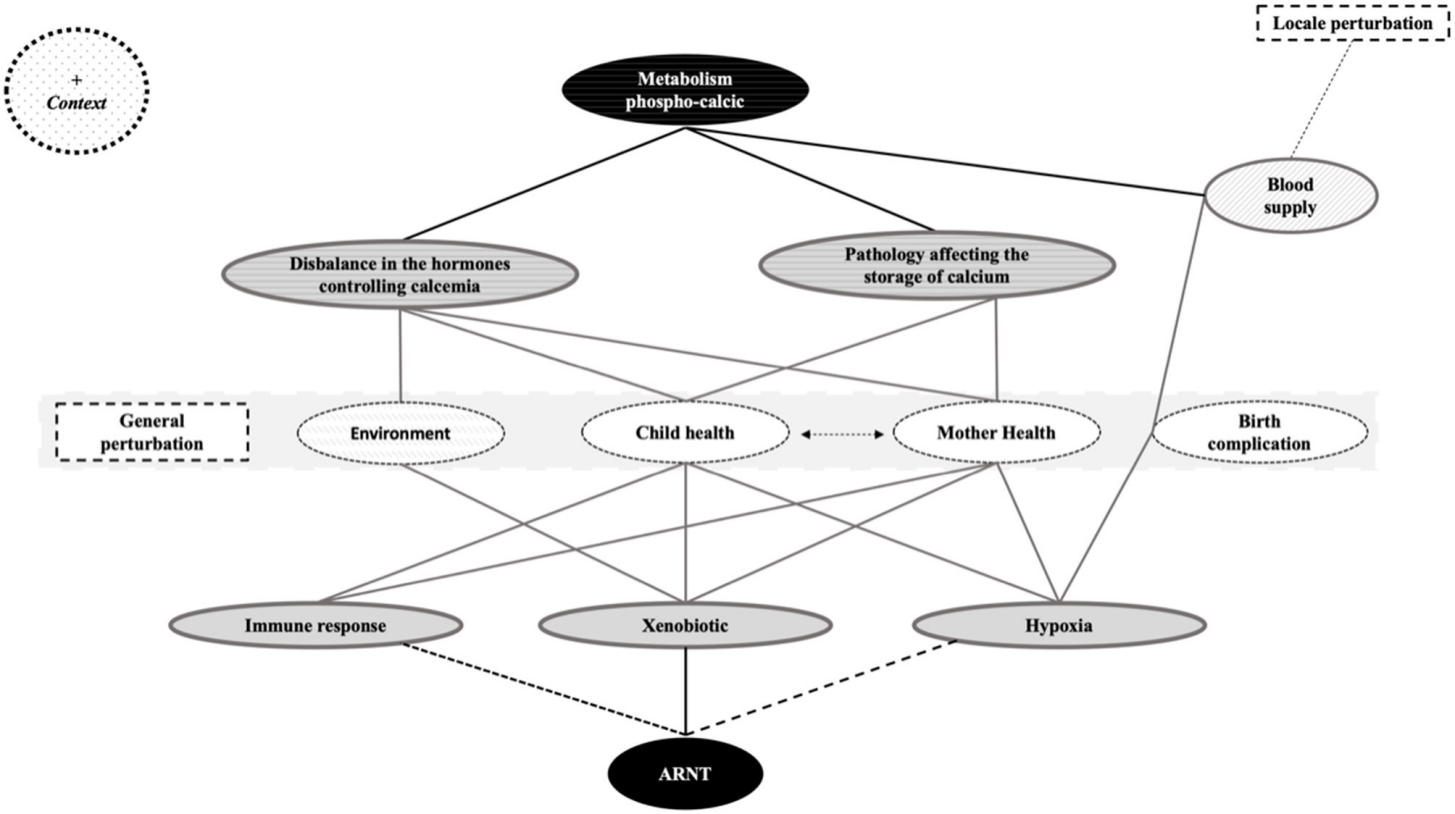
Identification of the different mechanisms showed by the clustering of aDDE risk and protector factors. ARNT, aryl hydrocarbon receptor nuclear translocator.

For the construction of common pathophysiological mechanisms, we looked at whether the 5 categories previously identified (Figure 3) were related to a general disruption (maternal health, infant health, birth complication, or impact or not by the environment) or to a local disruption. Figure 4 summarizes the 2 main pathways that will be modified upon exposure to these factors: the metabolism of calcium balance pathway and the aryl hydrocarbon receptor nuclear translocator (ARNT) pathway that is split into 3 mechanisms (response to hypoxia, response to xenobiotics, and immune response).

#### Metabolism of Calcium Balance

Tooth developments occur in three stages: a) ameloblast secretion (directly influenced by adequate levels of vitamin A, C, and D); b) mineralization; and c) maturing (directly affected by levels of calcium and phosphorus). An inadequate supply of calcium phosphate leads to a perturbation calcium phosphate deposit in the matrix (164) and deficiency of calcium can affect epithelial cell function and the mineralization process (165). During mineralization and maturation of enamel, many exchanges with the blood medium take place and involve the massive arrival of calcium and phosphate ions from the interstitial fluid.

Following this observation, the mechanisms disrupting calcium homeostasis could also disrupt enamel formation and the ameloblasts will not obtain the nutrients they need to secrete enamel (49). This observation will link perturbation of the blood supply of the developing tooth to its consequences for cellular nutrition, thus leading to aDDE. This explanation can also be applicable to exodontia and trauma. Globally (166), calcium and phosphate homeostasis is related to the levels of calcitriol (hormonally active form of vitamin D), of parathormone (PTH) and calcitonin. Organs involved in this process are the thyroid, bones, kidneys, and digestive tract. In this way, different groups of pathologies have an impact on enamel development:

1. Pathologies affecting the organs (digestive tract, bone, and kidney) responsible for storage of calcium.
2. An imbalance in the hormones controlling calcemia and phosphatemia, or a pathology of organs responsible for hormonal regulation (parathyroid and thyroid).

#### ARNT Pathway

The protein aryl hydrocarbon Receptor (AhR) is involved in the response to environmental pollutants (154). Its function is to regulate the expression of xenobiotic metabolizing enzymes involved in detoxication using the ARNT (167).

The conditions of hypoxia lead to reprogramming the cell via the Hypoxia-Inducible Factor (HIF) protein (168), who also required ARNT. These etiologies are different, but the molecular mechanisms involved seem to converge on a single path, as they use ARNT as a partner. At the cellular level, this could result in a disturbance of the normal ameloblastic activity (142, 164) and lead to disorders of dental enamel matrix protein. Vorrink and Domann (167) explained that the normal xenobiotic responses may be perturbed under physiological hypoxia, so the xenobiotic response and hypoxia response pathways intersect. The important role of ARNT in both the AhR and HIF signaling pathways establishes a meaningful foundation for a possible crosstalk between these two vitally important signaling pathways.

Because the AhR receptor is expressed on a majority of immune cell types, this reflects the importance of AhR in immunological processes (169). In addition, AhR signaling pathways have been reported to influence a few genes responsible for mediating inflammation and other immune responses, so an involvement of the immune response in the ARNT pathway is to be explored.

**Figure 5:**
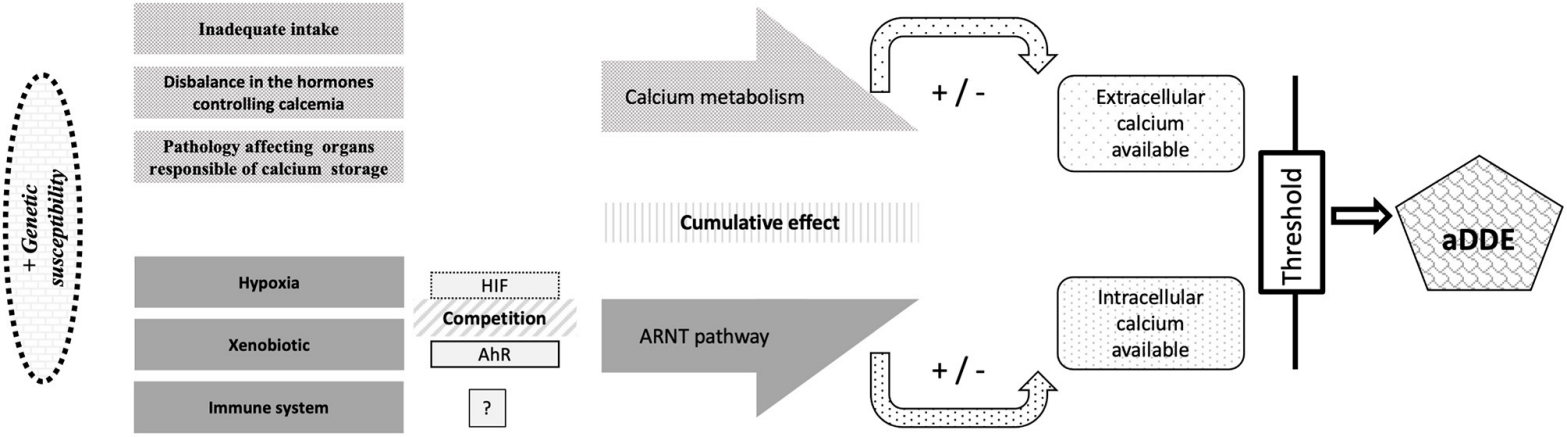
Proposed link between the mechanisms involved in aDDE formation and the cumulative and synergistic effect of the factors involved. HIF, Hypoxia-Inducible Factor; ARNT, aryl hydrocarbon receptor nuclear translocator; AhR, Aryl hydrocarbon Receptor; aDDE, aquired Developmental Defects of Enamel.

Skinner and Hung (94), postulated that a systemic event combined with a local traumatic event was a possible etiology of aDDE and Goho (170), reports that chemotherapy damage is directly related to dose (the ameloblast does not appear to be affected by a low dose of radiation, but that a high dose of radiation causes its death) and/or to the repetition of the various agents. This make us suggest that these mechanisms are cumulative and that when the equilibrium is destabilized, the other pathways adapt. However, there would be a threshold beyond which the organism cannot adapt and the very extensive clinical picture of aDDE raises the hypothesis of a host response (sex/age…) even an epigenetic control (171) although authors (172) report that it is likely that environmental factors exert a greater influence (Figure 5). We can suppose that the defects result from the nature and/or the accumulation of the aggressions (intensity, duration, and moment) but also from their interactions between them. Thus, we believe that the risk of developing enamel defects arises from a metabolic disorder or disruption involving the ARNT pathway that needs further study. But because many diseases can produce hypocalcemia, hypoxia and pyrexia (173) and that micronutrient deficiencies have been linked to compromised conception, gestation length, fetal development, and growth, potentially leading to pregnancy loss, preterm delivery, small birth size, birth defects, and long-term metabolic disruption (174), both pathways may be involved at the same time. Finally, because 2,3,7,8-Tetrachlorodibenzo-p-dioxin (TCDD) rapidly increases intracellular calcium concentrations via AhR pathways (175), it is possible that ARNT and metabolic balance are linked (Figure 5).

This proposal of pathophysiological mechanism is supported by the fact that some factors coexist: Dos Reis et al. (176) reports a higher prevalence of preterm baby and low birth weight observed in infants born to mothers with Acquired Immunodeficiency Syndrome (AIDS). de Oliveira et al. (13) reports that Children with congenital Zika syndrome had a higher frequency of problems related to breastfeeding and low weight compared with healthy children. Suckling et al. (61) reports that asthma may in part be associated with an increased frequency of, or susceptibility to viral infections. Otitis media is an infectious condition that could coexist with the presence of fever and use of medicines (27). Preterm and low birth weight children are at increased risk of lower respiratory tract infections (177). Maternal risk factors are numerous and some are also cited as risk factors for DDE: smoking, drug and alcohol use, medication consumption, maternal malnutrition, multiple births, congenital malformations, genital infections, unspecified bleeding, maternal age (under 17 and older than 34), and may result in intrauterine growth restriction and the infant being small for its gestational age (177). Grahnén et al.'s (129) study indicates that the common complications of pre-term birth such as asphyxia and/or hyperbilirubinemia seem to explain the higher incidence of aDDE in this children.

This taxonomy and the hypotheses related to the mechanism allow us to make, with caution (due to confounding factors in the interpretation of the different results), some recommendations:

Because differences in nutritional status, or obstetric and pediatric care may be important etiological factors for DDE, medical teams (dentists, midwives, obstetricians, pediatricians and others) should be aware that maternal health during pregnancy and infancy care is related to children's oral health. Optimal nutritional intakes during the pre- peri- and postnatal periods, careful handling of high fever, greater public awareness regarding misuse of drugs, education about xenobiotics and how to prevent DDE, are recommended to decrease its occurrence.

In view of our results, children with a complex health history (perinatal events or healthiness during childhood), with hypomineralized secondary primary molars (25, 48), and with a history of oral trauma should be considered at risk for DDE. Globally, the presence of DDE increases the risk of cavities and tooth wear because defective enamel is thinner, holds more plaque and is less resistant to dissolving in acid. Early detection of DDE is beneficial in establishing a prevention program to manage tooth sensitivity, cavities, and tooth wear (178). Children with DDE should be seen by a dentist as soon as the temporary teeth erupt and monitored on a regular basis as long as the permanent teeth erupt. Early management of these teeth allows a better long-term prognosis and quality of life.

If we extrapolate Murray and Johnsen's (179) results, the position and kind of defect on the enamel is useful to determine its origin and can help with a diagnosis. Therefore, since dental enamel tissue cannot remodel, anomaly of the teeth can represent a “past pathology map,” and DDE may indicate the time of insult to the developing fetus or infant. This allows for vigilance and motivation for early detection of disorders. Specifically, this can help with the diagnosis of deafness and neurological lesions (180), or may even allow early detection of children with learning problems (181). They can also, based on the observation that enamel defects may present among the first symptoms of celiac disease (70), the diagnosis of these oral manifestations might be helpful for an early diagnosis (69).

## Conclusion

DDE may result from a wide range of acquired etiological factors, 114 were listed and classified in this scoping review. The most frequently encountered pathologies are those causing a disorder of calcium homeostasis or a perturbation of the ARNT pathway in mother or child. The link between the ARNT pathway and metabolism deficiency in uncertain and needs to be defined. Furthermore, the role of this mechanism in tissue impairment is still unclear and needs to be explored.

This work confirmed that the child's general health, medical history, and oral health are intimately linked. Communication between medical teams is essential because some risk factors should alert health practitioners to set up a prevention of dental pathology, DDE can be a warning sign of general pathologies and modifying some behaviors could limit the risk of developing DDE. Finally, this scoping review may help the practitioner explaining to the patient the possible causes of his condition.

## Author Contributions

All the authors participated in drafting the manuscript, revising it critically, and approved the final version of the submitted manuscript.

## Conflict of Interest

The authors declare that the research was conducted in the absence of any commercial or financial relationships that could be construed as a potential conflict of interest.

## Publisher's Note

All claims expressed in this article are solely those of the authors and do not necessarily represent those of their affiliated organizations, or those of the publisher, the editors and the reviewers. Any product that may be evaluated in this article, or claim that may be made by its manufacturer, is not guaranteed or endorsed by the publisher.

## Abbreviations

AhR: protein aryl hydrocarbon Receptor
aDDE: acquired Developmental Defects of Enamel
ARNT: aryl hydrocarbon receptor nuclear translocator
DDE: Developmental Defects of Enamel
HIF: Hypoxia-Inducible Factor.
