# Supplementary material for: Factors and Mechanisms Involved in Acquired Developmental Defects of Enamel: A Scoping Review
Source: Front Pediatr. 2022 Feb 24;10:836708. doi: 10.3389/fped.2022.836708 (PMC8907975; doi:10.3389/fped.2022.836708)
Supplement: Supplementary file 2 [file Table_2.DOCX]

Online Supplement 2

- Search strategy

("develop"[All Fields] OR "develop"[All Fields] OR "developed"[All Fields] OR "developer"[All Fields] OR "developer s"[All Fields] OR "developers"[All Fields] OR "developing"[All Fields] OR "developments"[All Fields] OR "develops"[All Fields] OR "growth and development"[MeSH Subheading] OR ("growth"[All Fields] AND "development"[All Fields]) OR "growth and development"[All Fields] OR "development"[All Fields]) AND ("abnormalities"[MeSH Subheading] OR "abnormalities"[All Fields] OR "defects"[All Fields] OR "defect"[All Fields] OR "defect s"[All Fields] OR "defected"[All Fields] OR "defective"[All Fields] OR "defectively"[All Fields] OR "defectives"[All Fields]) AND ("dental enamel"[MeSH Terms] OR ("dental"[All Fields] AND "enamel"[All Fields]) OR "dental enamel"[All Fields] OR "enamel"[All Fields] OR "enamels"[All Fields] OR "enamel s"[All Fields] OR "enameled"[All Fields] OR "enameling"[All Fields] OR "enamelling"[All Fields]
