# Supplementary material for: Factors and Mechanisms Involved in Acquired Developmental Defects of Enamel: A Scoping Review
Source: Front Pediatr. 2022 Feb 24;10:836708. doi: 10.3389/fped.2022.836708 (PMC8907975; doi:10.3389/fped.2022.836708)
Supplement: Supplementary file 3 [file Table_3.docx]

|  | Paper | Study  Design | Country | Years enrollment | Years of examen or follow up | Teeth | | Age | Nb of participant | Factor | Quality Score |
| --- | --- | --- | --- | --- | --- | --- | --- | --- | --- | --- | --- |
|  |  |  |  |  |  | Primary | Permanent |  |  |  |  |
|  | Acar, S., Yetkıner, A.A., Ersın, N., Oncag, O., Aydogdu, S., Arıkan, C., 2012. Oral findings and salivary parameters in children with celiac disease: a preliminary study. Med. Princ. Pract. Int. J. Kuwait Univ. Health Sci. Cent. 21, 129–133. | Cross Sectional Study (CSS) | Turkey |  |  |  | X | 6-19 | 35/35 | Celiac | 1/8 |
|  | Alaluusua S, Lukinmaa PL, Koskimies M, et al. Developmental dental defects associated with long breast feeding. *Eur J Oral Sci*. 1996;104(5-6):493-497. | Case Control Study (CCS) | Finland | 1992-93 |  |  | X | 1) 7-8  2) 12 | 1) 40  2) 97 | Breastfeeding | 6/9 |
|  | Alaluusua S, Lukinmaa PL, Vartiainen T, Partanen M, Torppa J, Tuomisto J. Polychlorinated dibenzo-p-dioxins and dibenzofurans via mother’s milk may cause developmental defects in the child’s teeth. *Environ Toxicol Pharmacol*. 1996;1(3):193-197. | Cohort Study (CS) | Finland | 1987 | 6-7 years |  | X | 6-7 | 102 | Breasfeeding (Toxin) | 7/9 |
|  | Alaluusua S, Lukinmaa PL, Koskimies M, et al. Developmental dental defects associated with long breast feeding. *Eur J Oral Sci*. 1996;104(5-6):493-497. | CS | Italia |  |  |  | X | 24-35 | 48/65 | TCDD | 4/9 |
|  | Alexander, w.n., 1964. composite dysplasia of a single tooth as a result of electric burn damage: report of case. J. Am. Dent. Assoc. 1939 69, 589–591. | Case Report (CR) | United Stats |  |  |  | X | 21 | 1 | Electric Burn | 6/8 |
|  | Alpaslan, G., Alpaslan, C., Gögen, H., Oğuz, A., Cetiner, S., Karadeniz, C., 1999. Disturbances in oral and dental structures in patients with pediatric lymphoma after chemotherapy: a preliminary report. Oral Surg. Oral Med. Oral Pathol. Oral Radiol. Endod. 87, 317–321. | CS | Turkey |  | 3-58 month |  | X | 9-13 | 30/20 | Chemotherapy | 4/9 |
|  | Andreasen, J.O., Ravn, J.J., 1971. The effect of traumatic injuries to primary teeth on their permanent successors. II. A clinical and radiographic follow-up study of 213 teeth. Scand. J. Dent. Res. 79, 284–294. | CS | Denmark | 1955-66 | 1967-69 |  | X | 0-9 | 1003 | Traumatic injuries | 4/9 |
|  | Arrow, P., 2009. Risk factors in the occurrence of enamel defects of the first permanent molars among schoolchildren in Western Australia. Community Dent. Oral Epidemiol. 37, 405–415. | CS | Australia | 2005 | 2006-08 |  | X | 6-9 | 634 | Neonatal Health factor | 7/9 |
|  | Ashkenazi, M., Blumer, S., Eli, I., 2010. Effect of computerized delivery intraligamental injection in primary molars on their corresponding permanent tooth buds. Int. J. Paediatr. Dent. 20, 270–275. | CS | Israel | 1999-2007 | 0.5 to 8 years |  | X | at incluso. Mean 8.8 (+-2.45) at examination | 78 (166 teeth) | intraligamental injection  **No significant** | 5/9 |
|  | Atif M, Mathur VP, Tewari N, Bansal K, Rahul M, Bakhshi S. Long-Term Effect of Anticancer Therapy on Dentition in Childhood Cancer Survivors: An Observational, Cross-Sectional Study Indian J Pediatr. 2021 Jun 9. | CSS | India | 2017-19 | 2019 |  | X | >12 | 120/121 | Anticancer therapy befor 8 years | 6/8 |
|  | Avşar, A., Elli, M., Darka, O., Pinarli, G., 2007. Long-term effects of chemotherapy on caries formation, dental development, and salivary factors in childhood cancer survivors. Oral Surg. Oral Med. Oral Pathol. Oral Radiol. Endod. 104, 781–789. | CS | Turkey | 1999-2005 |  |  | X | 7-13 | 96/96 | Cancer | 5/9 |
|  | Bagattoni, S., D’Alessandro, G., Prete, A., Piana, G., Pession, A., 2014. Oral health and dental late adverse effects in children in remission from malignant disease. A pilot case-control study in Italian children. Eur. J. Paediatr. Dent. 15, 45–50. | CS | Italy | 2011-12 |  |  | X | 7-19 | 25/27 | Cancer | 5/9 |
|  | Balmer, R., Toumba, J., Godson, J., Duggal, M., 2012. The prevalence of molar incisor hypomineralisation in Northern England and its relationship to socioeconomic status and water fluoridation. Int. J. Paediatr. Dent. 22, 250–257. | CSS | England | 2008-09 |  |  | X | 12 | 3233 | Socioeconomic status  Fluorid water | 5/8 |
|  | Blanck-Lubarsch, M., Dirksen, D., Feldmann, R., Sauerland, C., Hohoff, A., 2019. Tooth Malformations, DMFT Index, Speech Impairment and Oral Habits in Patients with Fetal Alcohol Syndrome. Int. J. Environ. Res. Public. Health 16. | CS | Germany |  |  | X | X | 5-12 | 30/30 | Fœtal Alcohol syndrome | 5/9 |
|  | Bock JE, Winkel S. A follow-up study of infants who received intra-uterine transfusions because of severe rhesus haemolytic disease. *Acta Obstet Gynecol Scand Suppl*. 1976;53:37-40. | CS | Denmark |  |  | X | X | 5-91 month | 19 | Intra-uterin transfusion | 6/9 |
|  | Broadbent, J.M., Thomson, W.M., Williams, S.M., 2005. Does caries in primary teeth predict enamel defects in permanent teeth? A longitudinal study. J. Dent. Res. 84, 260–264. | CS | New Zeland | 1972-73 |  |  | X | 9 | 663 | decay | 8/9 |
|  | Cantekin, K., Gumus, H., Torun, Y.A., Sahin, H., 2015. The evaluation of developmental enamel defects and dental treatment conditions in a group of Turkish children with congenital heart disease. Cardiol. Young 25, 312–316. | CS | Turkey |  |  | X | X | 3-14 | 72/56 | Heart disese  **No significant** | 6/9 |
|  | Carpentier S, Ghijselings E, Schoenaers J, Carels C, Verdonck A. Enamel defects on the maxillary premolars in patients with cleft lip and/or palate: a retrospective case-control study. *Eur Arch Paediatr Dent Off J Eur Acad Paediatr Dent*. 2014;15(3):159-165. | CS | Belgium  Netherland | 1981-2001  1995-2000 |  |  |  |  | 123/181 | Cleft palat | 5/9 |
|  | Çetiner, D., Çetiner, S., Uraz, A., Alpaslan, G.H., Alpaslan, C., Toygar Memikoğlu, T.U., Karadeniz, C., 2019. Oral and dental alterations and growth disruption following chemotherapy in long-term survivors of childhood malignancies. Support. Care Cancer Off. J. Multinatl. Assoc. Support. Care Cancer 27, 1891–1899. | CS | Turkey |  |  |  | X | 4-13 | 53 | Chemotherapy | 7/9 |
|  | Cockburn, F., Belton, N.R., Purvis, R.J., Giles, M.M., Brown, J.K., Turner, T.L., Wilkinson, E.M., Forfar, J.O., Barrie, W.J., McKay, G.S., Pocock, S.J., 1980. Maternal vitamin D intake and mineral metabolism in mothers and their newborn infants. Br. Med. J. 281, 11–14. | Randomized Clinical Trial (RCT) | England |  |  | X |  | 2-6 | 506/633 pregant woman  61 child | Maternal Vit D | 2/10 |
|  | Corrêa-Faria, P., Martins-Júnior, P.A., Vieira-Andrade, R.G., Oliveira-Ferreira, F., Marques, L.S., Ramos-Jorge, M.L., 2013b. Developmental defects of enamel in primary teeth: prevalence and associated factors. Int. J. Paediatr. Dent. 23, 173–179. | CCS | Brazil | 2010 |  | X |  | 3-5 | 381 | Health | 4/9 |
|  | Corrêa-Faria, P., Martins-Júnior, P.A., Vieira-Andrade, R.G., Marques, L.S., Ramos-Jorge, M.L., 2013a. Perinatal factors associated with developmental defects of enamel in primary teeth: a case-control study. Braz. Oral Res. 27, 363–368. | CSS | Brazil | 2010 |  | X |  | 3-5 | 104/105 | Perinatal factor | 6/8 |
|  | Cortines, A.A. de O., Corrêa-Faria, P., Paulsson, L., Costa, P.S., Costa, L.R., 2019. Developmental defects of enamel in the deciduous incisors of infants born preterm: Prospective cohort. Oral Dis. 25, 543–549. | CS | Brazil | 2012-2013 | 24 month | X |  | 3-24 months | 54 | Preterm  Intubation | 4/9 |
|  | Dahllöf, G., Barr, M., Bolme, P., Modéer, T., Lönnqvist, B., Ringdén, O., Heimdahl, A., 1988. Disturbances in dental development after total body irradiation in bone marrow transplant recipients. Oral Surg. Oral Med. Oral Pathol. 65, 41–44. | CS | Sweden | 1978-83 |  |  | X | 6-15 | 16 | Bone marrow transplant and body irradiation | 5/9 |
|  | de Amorim, C.S., Americano, G.C.A., Moliterno, L.F.M., de Marsillac, M. de W.S., Andrade, M.R.T.C., Campos, V., 2018. Frequency of crown and root dilaceration of permanent incisors after dental trauma to their predecessor teeth. Dent. Traumatol. Off. Publ. Int. Assoc. Dent. Traumatol. 34, 401–405. | CS | Brazil | 2006-2016 |  |  | X | 0-9 | 588 teeth | Traumatic injuries to the primary dentition | 4/9 |
|  | de Oliveira AMM, de Melo EGM, Mendes MLT, et al. Oral and maxillofacial conditions, dietary aspects, and nutritional status of children with congenital Zika syndrome. *Oral Surg Oral Med Oral Pathol Oral Radiol*. 2020;130(1):71-77. | CSS | Brazil | 2018 |  | X |  | 16-18 month | 45/50 | Congenital Zika Syndrome | 5/8 |
|  | Dinur N, Becker T, Levin A, Zadik Y, Itzhak JB, Azizi H, Hadad A, Batashvili G, Solomonov M [Long-term dental implications of infant oral mutilation: a case series.](https://pubmed.ncbi.nlm.nih.gov/34561584/)Br Dent J. 2021 Sep;231(6):335-340 | CR | Israel | - | - |  | X |  | 12 | Infant oral mutilation | 4/8 |
|  | Dixon, D.A., 1968. Defects of structure and formation of the teeth in persons with cleft palate and the effect of reparative surgery on the dental tissues. Oral Surg. Oral Med. Oral Pathol. 25, 435–446. | CS | England |  |  | X | X | 4-16 | 100/200 | Cleft palate and surgery | 6/9 |
|  | Elfrink, M.E.C., Moll, H.A., Kiefte-de Jong, J.C., Jaddoe, V.W.V., Hofman, A., ten Cate, J.M., Veerkamp, J.S.J., 2014. Pre- and postnatal determinants of deciduous molar hypomineralisation in 6-year-old children. The generation R study. PloS One 9, e91057. | CS | Netherland | 2002-06 (Mother) | 2008-12 | X |  | 6 | 5697 | Pre- and Postnatal Determinants | 8/9 |
|  | Elger, W., Illge, C., Kiess, W., Körner, A., Kratzsch, J., Schrock, A., Hirsch, C., 2020. Relationship between deciduous molar hypomineralisation and parameters of bone metabolism in preschool children. Int. Dent. J. 70, 303–307. | CS | Germany | 2011-15 |  | X |  | 1-6 | 578 | Bone metabolism | 6/9 |
|  | Elzein R, Chouery E, Abdel-Sater F, Bacho R, Ayoub F. Molar-incisor hypomineralisation in Lebanon: association with prenatal, natal and postnatal factors. *Eur Arch Paediatr Dent Off J Eur Acad Paediatr Dent*. Published online September 5, 2020 | CCS | Lebanon |  |  |  | X | 7-9 | 659 | prenatal, natal and postnatal factor | 6/9 |
|  | Fagrell, T.G., Ludvigsson, J., Ullbro, C., Lundin, S.-A., Koch, G., 2011. Aetiology of severe demarcated enamel opacities--an evaluation based on prospective medical and social data from 17,000 children. Swed. Dent. J. 35, 57–67. | CCS | Sweden | 1997-99 |  |  | X | 9-11 | 595/1180 | prenatal, natal and postnatal factor | 5/9 |
|  | Ferrini, F.R.D., Marba, S.T.M., Gavião, M.B.D., 2008. Oral conditions in very low and extremely low birth weight children. J. Dent. Child. Chic. Ill 75, 235–242. | CS | Brazil | 2001-02 |  |  | X | 38-42 month | 52/52 | Birth Weight | 7/9 |
|  | França TKXS, Lima MDM, Lima CCB, Moura MS, Lopes TSP, Moura JSS, Moura LFAD. [Quilombola children and adolescents show high prevalence of developmental defects of enamel.](https://pubmed.ncbi.nlm.nih.gov/34231701/)  Cien Saude Colet. 2021 Jul;26(7):2889-2898. | CCS | Brazil | 2010 |  | X | X | 3-14 | 406 | Health | 4/8 |
|  | Franco, J., Maia, S.É., Vieira, A., Santos, C., Fonseca Silva, T., 2016. Dental manifestations of congenital syphilis. Int. J. Inf. Res. Rev. 3, 2620–2622. | CR | Brazil |  |  |  | X |  | 1 | Syphilis | 1/8 |
|  | Ghanim, A., Manton, D., Bailey, D., Mariño, R., Morgan, M., 2013. Risk factors in the occurrence of molar-incisor hypomineralization amongst a group of Iraqi children. Int. J. Paediatr. Dent. 23, | CSS | Irak |  |  |  | X | - | 823 | Risk factor | 6/8 |
|  | Ghapanchi, J., Kamali, F., Siavash, Z., HomanEbrahimi, Pourshahidi, S., Ranjbar, Z., 2015. The Relationship between Gestational Diabetes, Enamel Hypoplasia and DMFT in Children: A Clinical Study in Southern Iran. Br. J. Med. Med. Res. 10, 1–6 | CS | Iran | 2000-13 |  | X | X | 3-12 | 50/50 | Gestational Diabetes | 6/9 |
|  | Hall RK. Prevalence of developmental defects of tooth enamel (DDE) in a pediatric hospital department of dentistry population (1). *Adv Dent Res*. 1989;3(2):114-119. | CSS | Australia | 1960-87 |  | X | X | 2-19 | 7518/893 | Health | 3/8 |
|  | Holan, G., Topf, J., Fuks, A.B., 1992. Effect of root canal infection and treatment of traumatized primary incisors on their permanent successors. Endod. Dent. Traumatol. 8, 12–15. | CS | Israel |  |  |  | X | 8-12 | 29/29/59  teeth | Trauma + endodontic treatment | 5/9 |
|  | Hong, L., Levy, S.M., Warren, J.J., Bergus, G.R., Dawson, D.V., Wefel, J.S., Broffitt, B., 2004. Primary tooth fluorosis and amoxicillin use during infancy. J. Public Health Dent. 64, 38–44. | CS | United States | 1992-95 | 5 years | X |  | 5 | 490 | Antibiotic  Fluor | 7/9 |
|  | Hong, L., Levy, S.M., Warren, J.J., Broffitt, B., 2011. Amoxicillin Use during Early Childhood and Fluorosis of Later Developing Tooth Zones. J. Public Health Dent. 71, 229–235. | CS | United States |  | 32 month | X |  |  | 113/244 | Antibiotic  Fluor | 6/9 |
|  | Jackson, D., 1961. A clinical study of non-endemic mottling of enamel. Arch. Oral Biol. 5, 212–223. | CSS |  |  |  |  | X | 12 | 1040 | Health | 3/8 |
|  | Jackson, R.D., Kelly, S.A., Katz, B., Brizendine, E., Stookey, G.K., 1999. Dental fluorosis in children residing in communities with different water fluoride levels: 33-month follow-up. Pediatr. Dent. 21, 248–254. | CSS | United States |  | 1992-1994 |  | X | 7-14 | 701 | Fluo | 5/8 |
|  | Jacobsen, P.E., Haubek, D., Henriksen, T.B., Østergaard, J.R., Poulsen, S., 2014. Developmental enamel defects in children born preterm: a systematic review. Eur. J. Oral Sci. 122, 7–14. | CS | Denmark |  |  | X | X | 5-10 | 87/298 | Anti-Epileptic drugs | 8/9 |
|  | Jälevik, B., Norén, J., 2001. Enamel hypomineralization of permanent first molars: A morphological study and survey of possible aetiological factors. Int. J. Paediatr. Dent. Br. Paedodontic Soc. Int. Assoc. Dent. Child. 10, 278–89. | CSS | Sweden |  |  |  | X | 7-9 | 516 | Health | 6/8 |
|  | Jan, J., Vrbic, V., 2000. Polychlorinated biphenyls cause developmental enamel defects in children. Caries Res. 34, 469–473. | CS | Slovenia | 1997 |  |  | X | 8-14 | 202/202 | PCB | 7/9 |
|  | Johnsen, D., Krejci, C., Hack, M., Fanaroff, A., 1984. Distribution of enamel defects and the association with respiratory distress in very low birthweight infants. J. Dent. Res. 63, 59–64. | CS | United stats |  |  | X |  | 1-4 | 46/46 | Respiratory distress  Birth weight | 3/9 |
|  | Kaste, S.C., Hopkins, K.P., Bowman, L.C., Santana, V.M., 1998. Dental abnormalities in children treated for neuroblastoma. Med. Pediatr. Oncol. 30, 22–27. | CS | United stats | 1963-94 |  | X | X |  | 52 | Neuroblastoma | 5/9 |
|  | Khazaei Y, Harris CP, Heinrich J, Standl M, Kühnisch J. Association Study on Nutrition in the First Year of Life and Molar-Incisor Hypomineralization (MIH)-Results from the GINIplus and LISA Birth Cohort Studies. Int J Environ Res Public Health. 2021 Oct 29;18(21):11411. |  |  | 2010 |  |  | X | 10 | 1010 | Nutrition  No Significant |  |
|  | Korolenkova, M.V., Starikova, N.V., Udalova, N.V., 2019. The role of external aetiological factors in dental anomalies in non-syndromic cleft lip and palate patients. Eur. Arch. Paediatr. Dent. Off. J. Eur. Acad. Paediatr. Dent. 20, 105–111 | CS | Russia |  |  |  | X | 6-17 | 369/500 | Cleft palat | 5/9 |
|  | Lamm, C.I., Norton, K.I., Murphy, R.J., Wilkins, I.A., Rabinowitz, J.G., 1988. Congenital rickets associated with magnesium sulfate infusion for tocolysis. J. Pediatr. 113, 1078–1082. | CR | United States |  |  |  |  | Mother | 5 | Magnesium for Tocolytic | 4/8 |
|  | Lauritano, D., Petruzzi, M., 2012. Decayed, missing and filled teeth index and dental anomalies in long-term survivors leukaemic children: a prospective controlled study. Med. Oral Patol. Oral Cirugia Bucal 17, e977-980. | CS | Italy |  |  |  | X | 8-15 | 52/52 | Cancer | 6/9 |
|  | Lawson, B.F., Stout, F.W., Ahern, D.E., Sneed, W.D., 1971. The incidence of enamel hypoplasia associated with chronic pediatric lead poisoning. S. C. Dent. J. 29, 5–10. | CS | United States | 1960-70 |  |  | X | - | 22/32 | Lead | 4/9 |
|  | Lee, D.-W., Kim, Y.-J., Oh Kim, S., Choi, S.C., Kim, Jongbin, Lee, J.H., Kim, H.J., Shin, J., Lee, N.-Y., Kim, S.-M., Ra, J., Kim, Jihun, Yang, Y.-M., 2020. Factors Associated with Molar-Incisor Hypomineralization: A Population-Based Case-Control Study. Pediatr. Dent. 42, 134–140. | CCS | South Korea | 2017 |  | X | X | 6-13 | 524/524 | Risk factor associeted | 7/9 |
|  | Li Y, Navia JM, Bian JY. Prevalence and distribution of developmental enamel defects in primary dentition of Chinese children 3-5 years old. Community Dent Oral Epidemiol. 1995;23(2):72-79. | CSS | China | 1992 |  | X |  | 3-5 | 1344 | Nutrition | 4/8 |
|  | Lo, E.C.M., Zheng, C.G., King, N.M., 2003. Relationship between the presence of demarcated opacities and hypoplasia in permanent teeth and caries in their primary predecessors. Caries Res. 37, 456–461. | CS | China | 1992 | 2001 |  | X | 12 | 253 | Decay | 8/9 |
|  | Lopes-Fatturi, A., Menezes, J.V.N.B., Fraiz, F.C., Assunção, L.R. da S., de Souza, J.F., 2019. Systemic Exposures Associated with Hypomineralized Primary Second Molars. Pediatr. Dent. 41, 364–370. | CSS | Brazil | 2016 |  | X |  | 8 | 784 | Systemic Exposures | 7/8 |
|  | Lunardelli, S.E., Peres, M.A., 2006. Breast-feeding and other mother-child factors associated with developmental enamel defects in the primary teeth of Brazilian children. J. Dent. Child. Chic. Ill 73, 70–78. | CCS | Brazil |  |  | X |  | 3-5 | 102/113 | Maternal and child variable | 5/9 |
|  | Malanczuk, T., Opitz, C., Retzlaff, R., 1999. Structural changes of dental enamel in both dentitions of cleft lip and palate patients. J. Orofac. Orthop. Fortschritte Kieferorthopadie OrganOfficial J. Dtsch. Ges. Kieferorthopadie 60, 259–268 | CSS | Germany | 1987-89 |  | X | X | 1-19 | 1) 412  2) 22 | Cleft palat | 3/8 |
|  | Masterson, E.E., Fitzpatrick, A.L., Enquobahrie, D.A., Mancl, L.A., Conde, E., Hujoel, P.P., 2017. Malnutrition-related early childhood exposures and enamel defects in the permanent dentition: A longitudinal study from the Bolivian Amazon. Am. J. Phys. Anthropol. 164, 416–423. | CS | Bolivia | 2002 | 2015 |  | X | 10-17 | 337 | Malnutrition | 8/9 |
|  | Mastora, A., Vadiakas, G., Agouropoulos, A., Gartagani-Panagiotopoulou, P., Gemou Engesaeth, V., 2017. Developmental defects of enamel in first permanent molars associated with use of asthma drugs in preschool aged children: A retrospective case-control study. Eur. Arch. Paediatr. Dent. Off. J. Eur. Acad. Paediatr. Dent. 18, 105–111 | CCS | Greece | 2012-14 |  |  | X | 6-12 | 77/70 | Asthma drugs | 6/9 |
|  | McCormick J, Filostrat DJ. Injury to the teeth of succession by abscess of the temporary teeth. *J Dent Child*. 1967;34(6):501-504. | CS | United Stats | 1967 |  |  | X | - | 82-76 | Abscess | 6/9 |
|  | Mellander, M., Norén, J.G., Fredén, H., Kjellmer, I., 1982. Mineralization defects in deciduous teeth of low birthweight infants. Acta Paediatr. Scand. 71, 727–733. | CSS | Sweden | 1968-74 |  | X |  |  | 91/48 | Low birth weight  Seasonal variation | 7/8 |
|  | Memarpour, M., Golkari, A., Ahmadian, R., 2014. Association of characteristics of delivery and medical conditions during the first month of life with developmental defects of enamel. BMC Oral Health 14, 122 | CSS | Iran | 2013 |  |  | X | 9-11 | 974 | Delivery  Early medical condition | 6/8 |
|  | Muratbegovic, A., Markovic, N., Ganibegovic Selimovic, M., 2007. Molar incisor hypomineralisation in Bosnia and Herzegovina: aetiology and clinical consequences in medium caries activity population. Eur. Arch. Paediatr. Dent. Off. J. Eur. Acad. Paediatr. Dent. 8, 189–194 | CCS | Bosnia | 2004 |  |  | X | 12 | 138 | Child health | 5/9 |
|  | Needleman, H.L., Allred, E., Bellinger, D., Leviton, A., Rabinowitz, M., Iverson, K., 1992. Antecedents and correlates of hypoplastic enamel defects of primary incisors. Pediatr. Dent. 14, 158–166. | Fundamental Study (FS) |  | 1979-80 |  | X |  |  | 455 primary incisors (from 455 children) | Pre- peri- post natal | NA |
|  | Nelson, S., Albert, J.M., Lombardi, G., Wishnek, S., Asaad, G., Kirchner, H.L., Singer, L.T., 2010. Dental caries and enamel defects in very low birth weight adolescents. Caries Res. 44, 509–518. | CS | United Stats | 1996 | 14 years |  | X | 14 | 224 | Low birth weight | 7/9 |
|  | Neto MBC, Silva-Souza KP da, Maranhão VF, Botelho KVG, Heimer MV, Dos Santos-Junior VE. Enamel Defects in Deciduous Dentition and Their Association with the Occurrence of Adverse Effects from Pregnancy to Early Childhood. *Oral Health Prev Dent*. 2020;18(1):741-746. | CSS | Brazil | 2017 |  | X |  | 2-5 | 152 | Pregnacy  Child health | 6/8 |
|  | Ngoc, V.T.N., Huong, L.T., Van Nhon, B., Tan, N.T.M., Van Thuc, P., Hien, V.T.T., Dung, T.M., Van Toan, N., Anh, L.Q., Son, L.H., Chu-Dinh, T., Chu, D.-T., 2019. The higher prevalence of developmental defects of enamel in the dioxin-affected region than non-dioxin-affected region: result from a cross-sectional study in Vietnam. Odontology 107, 17–22. | CSS | Vietnam | 2015 |  |  |  | 18-45 | 2200 | Dioxin | 5/8 |
|  | Nirmala, S.V.S.G., Quadar, M.A., Veluru, S., Tharay, N., Kolli, N.K., Minor Babu, M.S., 2015. Apgar index as a probable risk indicator for enamel defects in primary dentition: a cross sectional study. J. Indian Soc. Pedod. Prev. Dent. 33, 229–233. | CSS | India |  |  | X |  | 4-5 | 108 | Apgar | 6/8 |
|  | Niswander, J.D., Sujaku, C., 1962. Relationship of enamel defects of permanent teeth to retention of deciduous tooth fragments. J. Dent. Res. 41, 808–814 | CSS | Japan | 1959 |  |  | X | 7-11 | 342 | Retention Deciduous tooth fragments | 3/8 |
|  | Noor Mohamed R, Basha S, Virupaxi SG, Idawara Eregowda N, Parameshwarappa P.[Hypomineralized Primary Teeth in Preterm Low Birth Weight Children and Its Association with Molar Incisor Hypomineralization-A 3-Year-Prospective Study.](https://pubmed.ncbi.nlm.nih.gov/34943307/)  Children (Basel). 2021 Dec 2;8(12):1111 | CSS | Saudi  Arabia | 2012-13 | 2016  2019 | X | X | 3,5-4,3  6,5-7,3 | 287/290 | Preterm  Low Birth Weight | 6/8 |
|  | Norén, J.G., 1984. Microscopic study of enamel defects in deciduous teeth of infants of diabetic mothers. Acta Odontol. Scand. 42, 153–156. | CS | Sweden | 1980-81 |  | X |  | - | 35 | Intubation | 5/9 |
|  | Nørrisgaard, P.E., Haubek, D., Kühnisch, J., Chawes, B.L., Stokholm, J., Bønnelykke, K., Bisgaard, H., 2019. Association of High-Dose Vitamin D Supplementation During Pregnancy With the Risk of Enamel Defects in Offspring: A 6-Year Follow-up of a Randomized Clinical Trial. JAMA Pediatr. | RCT | Danemark |  | **X** | **X** |  | pregant woman | 315/306 | Maternal vit D | 9/10 |
|  | Olczak-Kowalczyk, D., Kowalczyk, W., Krasuska-Sławińska, E., Dądalski, M., Kostewicz, K., Pawłowska, J., 2014. Oral health and liver function in children and adolescents with cirrhosis of the liver. Przeglad Gastroenterol. 9, 24–31. | CS | Poland |  |  | X | X | 1,5 -18 | 35 | Cirrhosis | 3/9 |
|  | Owosho, A.A., Brady, P., Wolden, S.L., Wexler, L.H., Antonescu, C.R., Huryn, J.M., Estilo, C.L., 2016. Long-term effect of chemotherapy-intensity-modulated radiation therapy (chemo-IMRT) on dentofacial development in head and neck rhabdomyosarcoma patients. Pediatr. Hematol. Oncol. 33, 383–392. | CS | United stats |  |  |  | X | 6-16 | 13 | Cancer therapy | 3/9 |
|  | Päivi Hölttä, Terttu Vartianen, 2001. Developmental Dental Defetcts in Childre who reside by a River Polluted by Dioxins and Furans. Arch. Environ. Health 522–527. | CSS | Finland | 1998-99 |  |  | X | 7-8 | 1030 | Dioxin and furans | 5/8 |
|  | Pajari, U., Lanning, M., Larmas, M., 1988. Prevalence and location of enamel opacities in children after anti-neoplastic therapy. Community Dent. Oral Epidemiol. 16, 222–226 | CS | Finland |  |  | X | X | 5-20 | 37/37 | Anti-neoplastic therapy | 6/9 |
|  | Pajari, U., Lanning, M., 1995. Developmental defects of teeth in survivors of childhood ALL are related to the therapy and age at diagnosis. Med. Pediatr. Oncol. 24, 310–314. | CS | Finland |  |  |  | X | Mean 12,5 | 45/45x2 | Leukemia | 7/9 |
|  | Pascon, T., Barbosa, A.M.P., Cordeiro, R.C.L., Bussaneli, D.G., Prudencio, C.B., Nunes, S.K., Pinheiro, F.A., Bossolan, G., Oliveira, L.G., Calderon, I.M.P., Marini, G., Rudge, M.V.C., 2019a. Prenatal exposure to gestational diabetes mellitus increases developmental defects in the enamel of offspring. PloS One 14, e0211771. | CS | Brazil | 2003-13 (Mother) | 2016-17 | X | X | 3-12 | 50/250 | Gestational diabetes | 8/9 |
|  | Pegelow, M., Alqadi, N., Karsten, A.L.-A., 2012. The prevalence of various dental characteristics in the primary and mixed dentition in patients born with non-syndromic unilateral cleft lip with or without cleft palate. Eur. J. Orthod. 34, 561–570. | CS | Sweden | 1986-97 |  | X | X | 5-10 | 129 | Cleft palat | 4/9 |
|  | Pimlott, J.F., Howley, T.P., Nikiforuk, G., Fitzhardinge, P.M., 1985. Enamel defects in prematurely born, low birth-weight infants. Pediatr. Dent. 7, 218–223. | CS | Canada |  |  | X | X | 1,5-8 | 106 | Low birth weight | 3/9 |
|  | Pindborg, J.J., 1969. Dental mutilation and associated abnormalities in Uganda. Am. J. Phys. Anthropol. 31, 383–389. | CSS | Uganda | 1966 |  |  | X | 0- +40 | 1169 | Mutilation | 3/8 |
|  | Pinho, J.R.O., Filho, F.L., Thomaz, E.B. a. F., Lamy, Z.C., Libério, S.A., Ferreira, E.B., 2012. Are low birth weight, intrauterine growth restriction, and preterm birth associated with enamel developmental defects? Pediatr. Dent. 34, 244–248. | CS | Brazil |  |  | X |  | 12-60 month | 205 | Low birth weight  Preterm | 8/9 |
|  | Pinho, J.R.O., Thomaz, E.B.A.F., Ribeiro, C.C.C., Alves, C.M.C., Silva, A.A.M. da, 2019. Factors associated with the development of dental defects acquired in the extrauterine environment. Braz. Oral Res. 33, e094. | CS | Brazil | 2010 | 2011-13 | X |  | 12-30 month | 982 | Child environment  Postnatal factor | 8/9 |
|  | Pinto, G.D.S., Costa, F.D.S., Machado, T.V., Hartwig, A., Pinheiro, R.T., Goettems, M.L., Demarco, F.F., 2018. Early-life events and developmental defects of enamel in the primary dentition. Community Dent. Oral Epidemiol. 46, 511–517. | CS | Brazil | 2009-11 | 2012-14 | X |  | 24-30 month | 503 | Early life events | 6/9 |
|  | Pitiphat, W., Luangchaichaweng, S., Pungchanchaikul, P., Angwaravong, O., Chansamak, N., 2014. Factors associated with molar incisor hypomineralization in Thai children. Eur. J. Oral Sci. 122, 265–270 | CSS | Thailand | 2011-12 |  |  | X | 7-8 | 420 | Pregnancy/delivery and Child health | 6/8 |
|  | Pontes, A.S., Lima, M. de D.M., Andrade, N.S., de Moura, M.S., Moura, L. de F.A. de D., 2017. Dental enamel development defects in children and adolescents with HIV infection: case-control study. Spec. Care Dent. Off. Publ. Am. Assoc. Hosp. Dent. Acad. Dent. Handicap. Am. Soc. Geriatr. Dent. 37, 19–27. | CS | Brazil |  |  | X | X | 3-15 | 52/104 | HIV | 4/9 |
|  | Purvis RJ, Barrie WJ, MacKay GS, Wilkinson EM, Cockburn F, Belton NR. Enamel hypoplasia of the teeth associated with neonatal tetany: a manifestation of maternal vitamin-D deficiency. *Lancet Lond Engl*. 1973;2(7833):811-814. | CS | Scotland | 1966-69 |  | X |  | 42 month (mean) | 49 | Maternal vitamin D | 6/9 |
|  | Rai A, Singh A, Menon I, Singh J, Rai V, Aswal GS. Molar Incisor Hypomineralization: Prevalence and Risk Factors Among 7-9 Years Old School Children in Muradnagar, Ghaziabad. *Open Dent J*. 2018;12:714-722. | CSS | India | 2014-15 |  |  | X | 7-9 | 992 | Pregnancy/delivery and Child health | 7/8 |
|  | Rattner, L.J., Myers, H.M., 1962. Occurrence of enamel hypoplasia in children with congenital allergies. J. Dent. Res. 41, 646–649. | CS | England |  |  | X | X | 3-9 | 45 | Allergies | 3/9 |
|  | Ravn, J.J., 1975. Developmental disturbances in permanent teeth after exarticulation of their primary predecessors. Scand. J. Dent. Res. 83, 131–134. | CS | Denmark |  |  | X |  | - | 77 | Exarticulation | 3/9 |
|  | Reed, S.G., Voronca, D., Wingate, J.S., Murali, M., Lawson, A.B., Hulsey, T.C., Ebeling, M.D., Hollis, B.W., Wagner, C.L., 2017. Prenatal vitamin D and enamel hypoplasia in human primary maxillary central incisors: a pilot study. Pediatr. Dent. J. Int. J. Jpn. Soc. Pediatr. Dent. 27, 21–28. | RCT | United Stats |  |  | X |  |  | 37 children | Maternal vit D | 2/10 |
|  | Reed, S.G., Miller, C.S., Wagner, C.L., Hollis, B.W., Lawson, A.B., 2020. Toward Preventing Enamel Hypoplasia: Modeling Maternal and Neonatal Biomarkers of Human Calcium Homeostasis. Caries Res. 54, 55–67. | RCT | United Stats | 2004-2009 Pregnancy |  | X |  |  | 350 Mothers  145 Children | Calcium Homeostasis | 1/10 |
|  | Reis CLB, Barbosa MCF, de Lima DC, Brancher JA, Lopes CMCF, Baratto-Filho F, Küchler EC, de Oliveira DSB. Risk factors for developmental defects of enamel in children from southeastern Brazil. Community Dent Health. 2021 Aug 31;38(3):178-181 | CSS | Brazil | 2018 |  | X | X | 8-11 | 353 | Low birth | 5/8 |
|  | Rugg-Gunn, A.J., Al-Mohammadi, S.M., Butler, T.J., 1998. Malnutrition and developmental defects of enamel in 2- to 6-year-old Saudi boys. Caries Res. 32, 181–192. | CSS | Saudi-Arabia | 1993/94 |  | X |  | 2-6 | 390 | Malnutrition | 5/8 |
|  | Rule, J.T., Zacherl, W.A., Pfefferle, A.M., 1972. The relationship between ankylosed primary molars and multiple enamel defects. ASDC J. Dent. Child. 39, 29–35 | CS | United states | - | - |  | X | 6,5-14,5 | 23/23 | Ankylosis | 4/9 |
|  | Saitoh, M., Nakamura, Y., Hanasaki, M., Saitoh, I., Murai, Y., Kurashige, Y., Fukumoto, S., Asaka, Y., Yamada, M., Sekine, M., Hayasaki, H., Kimoto, S., 2018. Prevalence of molar incisor hypomineralization and regional differences throughout Japan. Environ. Health Prev. Med. 23, 55. | CSS | Japan | 2015-16 |  |  | X | 7-9 | 4985 | Regional Residancy | 5/8 |
|  | Schüler, I.M., Haberstroh, S., Dawczynski, K., Lehmann, T., Heinrich-Weltzien, R., 2018. Dental Caries and Developmental Defects of Enamel in the Primary Dentition of Preterm Infants: Case-Control Observational Study. Caries Res. 52, 22–31 | CS | Germany | 2014-15 |  | X |  | 3-4 | 64/43 | Preterm | 8/9 |
|  | Schüttfort G, Höfler S, Kann G, et al. Influence of tenofovir exposure in utero on primary dentition. *Eur J Pediatr*. 2020 |  | Germany | 2016-17 (mother) |  | X |  | 2 | 31 | Tenoferovir  **No significant** | 3/9 |
|  | Sevbitov, A., Kuznetsova, M., Dorofeev, A., Borisov, V., Mironov, S., Iusupova, I., 2020. Dental anomalies in people living in radionuclide-contaminated regions. J. Environ. Radioact. 216, 106190. | CSS | Russia  Poland |  |  | X | X | 3-44 | 1889 | Radio active contamination | 5/8 |
|  | Shteyer, E., Berson, T., Lachmanovitz, O., Hidas, A., Wilschanski, M., Menachem, M., Shachar, E., Shapira, J., Steinberg, D., Moskovitz, M., 2013. Oral health status and salivary properties in relation to gluten-free diet in children with celiac disease. J. Pediatr. Gastroenterol. Nutr. 57, 49–52. | CS | Israel |  |  | X | X | 1-18 | 30/30/30 | Celiac disease | 4/9 |
|  | Silva, A., Bravo-González, L.A., Vicente, A., 2020. Second primary molar hypomineralisation and drugs used during pregnancy and infancy. A systematic review. Clin. Oral Investig. 24, 1287–1297 | CS | Australia | 2007 | 2014-16 | X |  | 6 | 344 | Child health  Mother habits | 5/9 |
|  | Silva MCPM da, Arnaud M de A, Lyra MCA, et al. Dental development in children born to Zikv-infected mothers: a case-based study. *Arch Oral Biol*. 2020;110:104598. | CS | Brazil | 2015 | 3 years | X |  | 6-36 month | 13 | Zika | 4/9 |
|  | Skaare, A.B., Aas, A.-L.M., Wang, N.J., 2015. Enamel defects on permanent successors following luxation injuries to primary teeth and carers’ experiences. Int. J. Paediatr. Dent. 25, 221–228. | CS | Norway | 2003 | 2010 |  | X | 8-15 | 193 | Local Trauma | 6/9 |
|  | Skinner, M.F., Hung, J.T., 1989. Social and biological correlates of localized enamel hypoplasia of the human deciduous canine tooth. Am. J. Phys. Anthropol. 79, 159–175. | CCS | Canada | 1985-86 |  | X |  | 4-6 | 33/86 | Calcium  Local Trauma | 6/9 |
|  | Sonis, A.L., Tarbell, N., Valachovic, R.W., Gelber, R., Schwenn, M., Sallan, S., 1990. Dentofacial development in long-term survivors of acute lymphoblastic leukemia. A comparison of three treatment modalities. Cancer 66, 2645–2652. | CS | United states | 1973-83 |  |  | X | 8-16 | 97 | Cancer treatment | 3/9 |
|  | Spitzer, R., 1942. Enamel Hypoplasia in Idiopathic Epilepsy. Br. Med. J. 1, 110. | CSS | Germany |  |  |  | X |  | 33 | Idiopatic Epilepsy | 3/8 |
|  | Stagno, S., Pass, R.F., Thomas, J.P., Navia, J.M., Dworsky, M.E., 1982. Defects of tooth structure in congenital cytomegalovirus infection. Pediatrics 69, 646–648. | CS | United States | Birth |  | X |  | >1,5 | 118 | CMV | 5/9 |
|  | Stein, G., 1947. Enamel damage of systemic origin in premature birth and diseases of early infancy. Am. J. Orthod. 33, 831–841 | FS | United States |  |  | X |  |  | 60/Several hundred | Prematurity  Health | NA |
|  | Stimmler, L., Snodgrass, G.J., Jaffe, E., 1973. Dental defects associated with neonatal symptomatic hypocalcaemia. Arch. Dis. Child. 48, 217–220. | CS | England |  |  | X |  | 1,5-4 | 12 | Hypocalcemia | 3/9 |
|  | Subramaniam, P., Gupta, M., Mehta, A., 2012. Oral health status in children with renal disorders. J. Clin. Pediatr. Dent. 37, 89–93. | CS | India |  |  | X | X | 4-14 | 36 | Renal disorders | 4/9 |
|  | Suckling, G.W., Herbison, G.P., Brown, R.H., 1987. Etiological factors influencing the prevalence of developmental defects of dental enamel in nine-year-old New Zealand children participating in a health and development study. J. Dent. Res. 66, 1466–1469. | CS | New-Zeland | 1972-1973 |  |  |  | 9 | 696 | Health | 8/9 |
|  | Suely Falcao de Oliveira Melo, N., Guimaraes Vieira Cavalcante da Silva, R.P., Adilson Soares de Lima, A., 2014. The neonatal intubation causes defects in primary teeth of premature infants. Biomed. Pap. Med. Fac. Univ. Palacky Olomouc Czechoslov. 158, 605–612. | CS | Brazil | 2008-10 |  | X |  | 2 | 157 | Intubation | 7/9 |
|  | Sweeney, E.A., Cabrera, J., Urrutia, J., Mata, L., 1969. Factors associated with linear hypoplasia of human deciduous incisors. J. Dent. Res. 48, 1275–1279. | CS | Guatemala | 1964-66 |  | X |  | 1,5-2,5 | 73 | Infection | 6/9 |
|  | Takaoka, L.A.M.V., Goulart, A.L., Kopelman, B.I., Weiler, R.M.E., 2011. Enamel defects in the complete primary dentition of children born at term and preterm. Pediatr. Dent. 33, 171–176 | CS | Brazil | 2005-07 |  | X |  | 3-4 | 45/46 | Pre-term | 4/9 |
|  | Tapias-Ledesma, M.A., Jiménez, R., Lamas, F., González, A., Carrasco, P., Gíl de Miguel, A., 2003. Factors associated with first molar dental enamel defects: a multivariate epidemiological approach. J. Dent. Child. Chic. Ill 70, 215–220. | CCS | Spain | March to May 2000 |  |  | X | 9-11 | 48/149 | Health/drugs | 5/9 |
|  | Tariq, A., Alam Ansari, M., Owais Ismail, M., Memon, Z., 2014. Association of the use of bacterial cell wall synthesis Inhibitor drugs in early childhood with the Developmental Defects of Enamel. Pak. J. Med. Sci. 30, 393–397. | CSS | Pakistan |  |  |  | X | 7-14 | 367 | Antibiotic | 4/8 |
|  | Thomaz ÉBAF, Alves CMC, Ribeiro CCC, et al. Perinatal outcomes and changes in the oral cavity: Brazilian cohorts of Ribeirão Preto and São Luís. *Rev Bras Epidemiol Braz J Epidemiol*. 2015;18(4):966-970. | CS | Brazil |  |  | X |  | 13-30 months |  | Perinatal outcomes | 5/9 |
|  | Velló, M.A., Martínez-Costa, C., Catalá, M., Fons, J., Brines, J., Guijarro-Martínez, R., 2010. Prenatal and neonatal risk factors for the development of enamel defects in low birth weight children. Oral Dis. 16, 257–262. | CS | Spain |  |  | X |  | 4-5 | 52/50 | Low birth weight | 5/9 |
|  | Venkatesh Babu, N.S., Patel, P.B., 2016. Oral health status of children suffering from thyroid disorders. J. Indian Soc. Pedod. Prev. Dent. 34, 139–144. | CS | India |  | X | X |  | 2-16 | 200 | Thyroid disorder | 6/9 |
|  | Via WF, Churchill JA. Relationship of enamel hypoplasia to abnormal events of gestation and birth. *J Am Dent Assoc 1939*. 1959;59:702-707. | CSS |  |  |  | X |  | 2-6 | 312 | Gestation-birth events | 3/8 |
|  | Wagner, Y., 2016. Developmental defects of enamel in primary teeth - findings of a regional German birth cohort study. BMC Oral Health 17, 10. | CS | Germany | 2009-10 | 3 years | X |  | 3 | 377 | Pre term  Hospitalisation in the first year of life | 7/9 |
|  | Williamson, J.J., 1966. Trauma during exodontia. An aetiologic factor in hypoplastic premolars. Br. Dent. J. 121, 284–289. | CR |  |  |  |  | X |  | 10 | Exodontia | 3/8 |
|  | Wong HM, Peng S-M, King NM, McGrath C. Infant Growth and the Occurrence of Developmental Defects of Enamel in 12-Year-Olds. *Caries Res*. 2015;49(6):575-582. | CSS | China | 1997 | 2010 |  | X | 13 | 485 | Heavy birth weight  Rapid growth | 7/8 |
|  | Wong HM. Aetiological Factors for Developmental Defects of Enamel. *Austin J Anat.* 2014:1003. | CS | China | 1997 | 2010 |  | X | 12 | 514 | Sever disease | 7/9 |
|  | Wuollet, E., Laisi, S., Salmela, E., Ess, A., Alaluusua, S., 2014. Background factors of molar-incisor hypomineralization in a group of Finnish children. Acta Odontol. Scand. 72, 963–969. | CSS | Finland |  |  |  | X | 7-13 | 818 | -- | 6/8 |
